# Plus-strand RNA viruses hijack Musashi homolog 1 to shield viral RNA from cytoplasmic ribonuclease degradation

**DOI:** 10.1128/jvi.00023-25

**Published:** 2025-02-12

**Authors:** Defang Zhou, Menglu Xu, Qingjie Liu, Ruixue Xin, Gege Cui, Longying Ding, Xiaoyang Liu, Xinyue Zhang, Tianxing Yan, Jing Zhou, Shuhai He, Liangyu Yang, Bin Xiang, Ziqiang Cheng

**Affiliations:** 1 College of Veterinary Medicine, Shandong Agricultural University 34734 , Tai'an, Shandong, China; 2 College of Animal Science and Technology, Xinyang Agriculture and Forestry University 384191 , Xinyang, Henan, China; 3 College of Veterinary Medicine, Yunnan Agricultural University 12616 , Kunming, Yunnan, China; St. Jude Children's Research Hospital, Memphis, Tennessee, USA

**Keywords:** plus-strand RNA virus, MSI1, ribonuclease, viral RNA degradation, RNA-binding protein

## Abstract

A successful strategy employed by RNA viruses to achieve replication is to evade host cell RNase degradation. However, the mechanisms through which plus-strand RNA viruses effectively shield viral RNA from cellular ribonuclease degradation remain unclear. In this study, we identified the phenomenon whereby plus-strand RNA viruses, including avian leukosis virus subgroup J (ALV-J), reticuloendotheliosis virus (REV), chicken astrovirus (CAstV), and porcine epidemic diarrhea virus (PEDV), hijacked host cellular Musashi homolog 1 (MSI1). These viruses upregulated MSI1 expression and facilitated its translocation from the cytoplasmic periphery to a position proximal to and within the nucleus, thereby protecting viral RNA from degradation. Mechanistic analyses revealed that these viruses use distinct regions, the unique (U3) region or three prime untranslated region (3′UTR), to engage with MSI1, consequently shielding their viral RNA from cytoplasmic ribonuclease degradation. These results offer significant implications for understanding the replication tactics used by plus-strand RNA viruses, thereby advancing our understanding of their biological behaviors.

**IMPORTANCE:**

The intricate interplay between RNA viruses and host cell RNA regulation encompasses viral mechanisms designed to circumvent RNase-mediated degradation. However, the specific strategies employed by plus-strand RNA viruses to shield their RNA from host ribonucleases remain inadequately characterized. In this study, Musashi homolog 1 (MSI1) is predominantly localized in the cytoplasm of normal cells, distinct from the nucleus. Following infection by plus-strand RNA viruses such as avian leukosis virus subgroup J (ALV-J), reticuloendotheliosis virus (REV), chicken astrovirus (CAstV), and porcine epidemic diarrhea virus (PEDV), these viruses hijack MSI1 to relocate near and within the nucleus. This hijacking is facilitated by specific regions, including unique or three prime untranslated regions, thereby preventing viral RNA from degradation by cytoplasmic ribonucleases. These findings have significant implications for elucidating the replication strategies of plus-strand RNA viruses, thereby advancing our understanding of their biological mechanisms.

## INTRODUCTION

Viral infections are influenced by a complex set of interactions between viruses and their hosts (1–3). Notably, numerous viruses have evolved diverse mechanisms to evade the cellular ribonuclease (RNase) machinery responsible for the prompt degradation of foreign viral RNA upon entry into host cells. Specific structural modifications enable viral RNA to evade cellular RNase recognition, thereby altering their chemistry and reducing their susceptibility to binding and degradation (4, 5). However, viruses may disrupt the activity of cellular RNases through the release of inhibitory factors, thus acquiring temporal and spatial advantages for their replication (6, 7). Viral RNA can also form stable complexes with specific proteins within the host cell, further shielding it from attack by cellular RNase (8).

These stable complexes play crucial roles in the viral life cycle, and their formation often involves the interaction with specific host cell proteins. One such significant protein is the RNA-binding protein (RBP), which has drawn extensive research attention. For instance, Musashi homolog 1 (MSI1) is a highly conserved and ubiquitously expressed RBP that was initially discovered in the central nervous system (8, 9). MSI1 consists of two RNA recognition motifs (RRMs), RRM1 and RRM2, that are linked by a short linker in the N-terminal region, followed by an intrinsically disordered C-terminal region (10). The MSI1 binding site (MBS) has been extensively characterized as a single-stranded RNA motif containing the consensus sequence A/GU(1-3)AG, and MSI1 has been shown to selectively target the MBS within the untranslated region (UTR) of Zika virus to facilitate viral replication (11–13). Recent studies have demonstrated that avian leukosis virus subgroup J (ALV-J) and reticuloendotheliosis virus (REV) can enhance MSI1 expression, and conserved MBS can be found in the genomes of these viruses (14). Therefore, investigating the indispensability of MSI1 during ALV-J and REV replication is valuable. Considering their classification as plus-strand RNA viruses, such as the Zika virus, it is imperative to further investigate the role of MSI1 in the replication of other plus-stranded RNA viruses. RBPs are a class of critical host components that are hijacked by RNA viruses and regulate viral replication (15, 16). Emerging evidence has shown that some host RBPs enhance viral RNA template selection by selectively binding to specific cis-acting elements in the viral UTR (16, 17). Furthermore, co-opted host RBPs in the 5′-end of the viral genome potentially initiate viral RNA synthesis (18). Viruses also interact with cellular RBPs to maintain viral RNA integrity and block RNA degradation pathways (19–21). However, despite these findings, the mechanism underlying the promotion of viral replication by the binding of MSI1 to the viral MBS remains unclear.

In this study, we found that various plus-strand RNA viruses, including ALV-J, REV, chicken astrovirus (CAstV), and porcine epidemic diarrhea virus (PEDV), hijack MSI1 to prevent the degradation of viral RNAs. MBSs are located within the 3′UTRs or unique (U3) regions of these viral RNA genomes. Mechanistically, we revealed that these plus-strand RNA viruses utilized their U3 regions or 3′UTRs to bind with MSI1, thereby hijacking MSI1 and consequently safeguarding their viral RNA from degradation by host RNases in the cytoplasm. Our findings shed new light on the replication mechanisms of plus-strand RNA viruses.

## RESULTS

### MSI1 facilitates the replication of CAstV and PEDV by interacting with their 3′UTR

**Fig 1:**
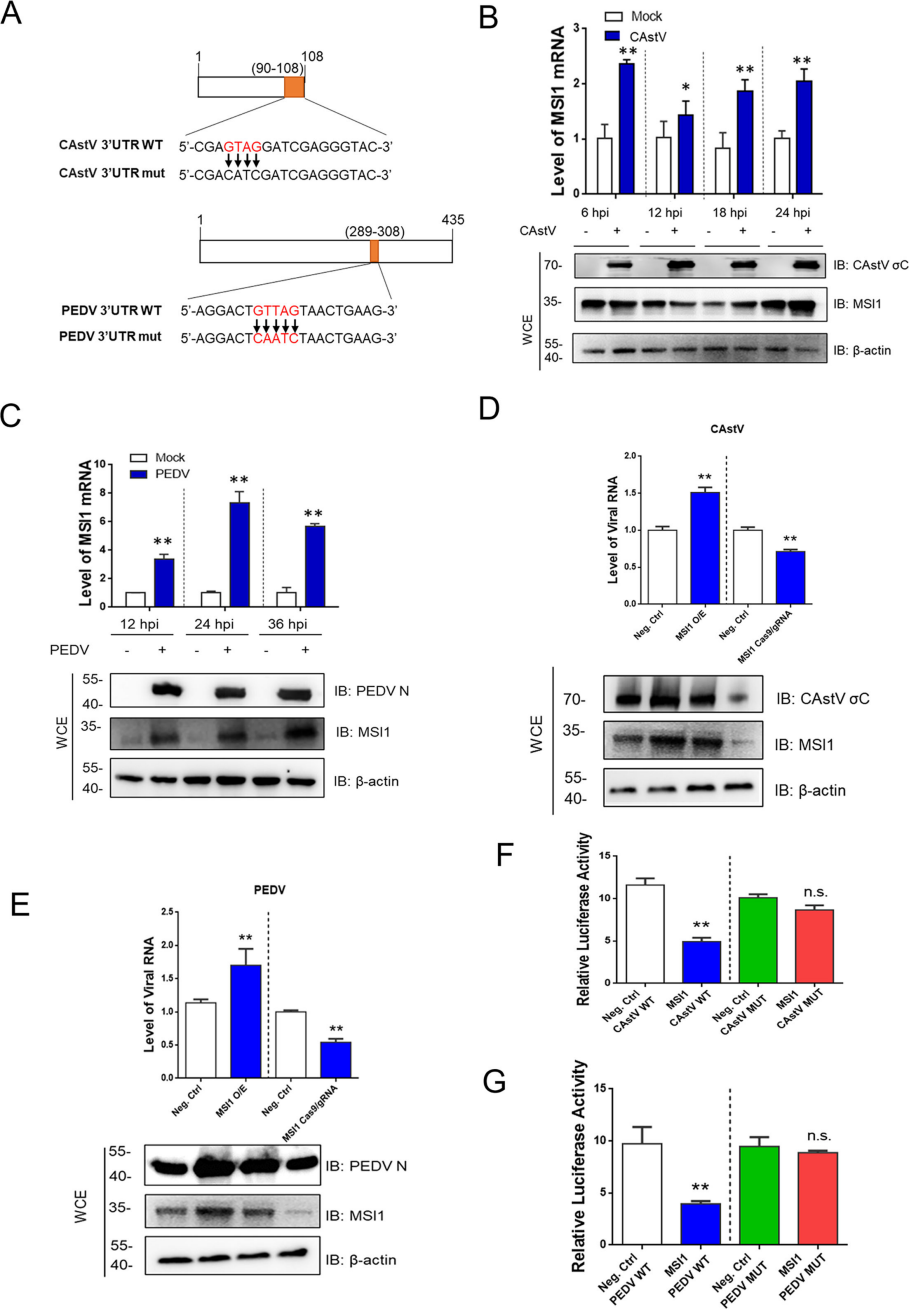
MSI1 is required for the replication of CAstV and PEDV by interacting with their 3′UTR. (**A**) The qRT-PCR and WB results demonstrated that CAstV δC protein increased the expression of MSI1 at a multiplicity of infection (MOI) of 0.1 at 8 hpi, 12 hpi, 18 hpi, and 24 hpi in LMH cells (avian leghorn male hepatoma cell line). (**B**) The qRT-PCR and WB results demonstrated that PEDV N protein increased the expression of MSI1 at an MOI of 0.1 at 12 hpi, 24 hpi, and 36 hpi in Vero cells. (**C**) The qRT-PCR and WB results showed that MSI1 overexpression promoted CAstV δC RNA and protein levels at 48 hpi, and MSI1 knockdown decreased CAstV RNA and protein levels at 48 hpi. (**D**) The qRT-PCR and WB results showed that MSI1 overexpression promoted PEDV N RNA and protein levels, and MSI1 knockdown decreased PEDV RNA and protein levels. (**E**) Bioinformatics analysis identified one putative MSI1 binding site at the CAstV and the PEDV 3ʹUTRs. (**F**) Luciferase reporter assays revealed that MSI1 significantly inhibited the activity of the CAstV 3′UTR reporter. (**G**) Luciferase reporter assays revealed that MSI1 significantly inhibited the activity of the PEDV 3′UTR reporter. Data are presented as mean ± SEM for *n* = 3, with each experiment being performed in triplicates. **P ≤ 0.01 by Student’s *t*-test versus the negative control group. n.s., not significant.

Bioinformatics analysis revealed that analogous MBS sequences are located in the 3′UTRs of other positive-sense RNA viruses, including CAstV and PEDV. This analysis was conducted using various software tools and online resources, such as RNA22, NCBI, ENSEMBLE, and the RNA–protein interaction prediction platform (http://pridb.gdcb.iastate.edu/RPISeq/#opennewwindow) (Fig. 1A). Consequently, we focused on examining the expression of MSI1 during infection with CAstV and PEDV. The results of quantitative reverse transcription polymerase chain reaction (qRT-PCR) and Western blot (WB) demonstrated that CAstV and PEDV increased the expression of MSI1 (Fig. 1B and C). MSI1 overexpression increased CAstV and PEDV RNA and protein levels, and MSI1 knockdown decreased their RNA and protein levels (Fig. 1D and E). Dual-luciferase assays were performed in 293T cells to confirm that MSI1 directly targeted CAstV and PEDV 3′UTR. Luciferase reporter assays revealed that MSI1 significantly inhibited the activity of the CAstV and PEDV 3′UTR reporters but not that of the control reporters (Fig. 1F and G). Mutations in the putative MSI1 binding site eliminated the MSI1 inhibitory effect on reporter activities of the PEDV and astrovirus 3′UTRs. These findings suggest that CAstV and PEDV hijack MSI1 for the viral replication of CAstV and PEDV through its interaction with their 3′UTR, respectively.

### MSI1 maintains the replication of ALV-J and REV by targeting viral U3s

**Fig 2:**
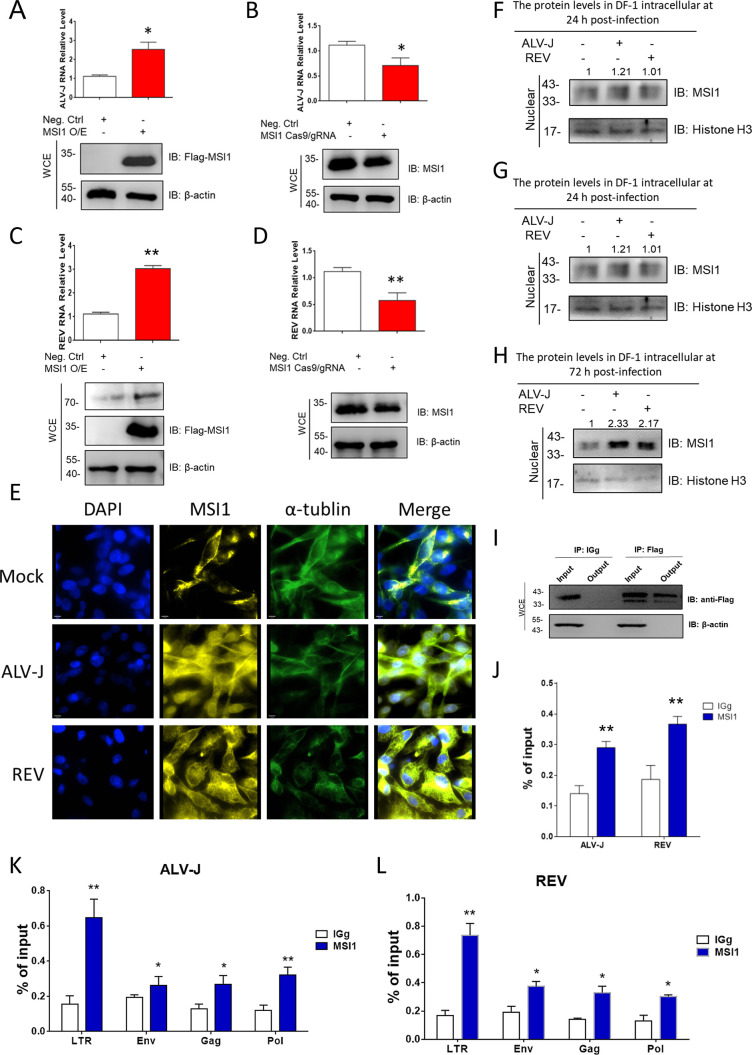
MSI1 is required for the replication of ALV-J and REV by binding U3s. (**A**) MSI1 overexpression activated ALV-J replication in ALV-J infection at an MOI of 0.01 at 48 hpi, as detected using WB and qRT-PCR. (**B**) MSI1 knockdown inhibited ALV-J replication in ALV-J infection at an MOI of 0.01 at 48 hpi, as detected using WB and qRT-PCR. (**C**) MSI1 overexpression activated REV replication in REV mono-infection at an MOI of 0.01 at 48 hpi, as detected using WB and qRT-PCR. (**D**) MSI1 knockdown inhibited REV replication in REV infection at an MOI of 0.01 at 48 hpi, as detected using WB and qRT-PCR. qRT-PCR data are presented as mean ± SEM and were obtained from three independent experiments (*n* = 3); each experiment contained triplicates. (**E**) CLSM was used to observe the localization and expression of MSI1 in normal DF-1 cells, ALV-J-infected, and REV-infected DF-1 cells at 72 hpi, as detected with an anti-Flag antibody, alpha-tubulin monoclonal antibody. Nuclei are labeled with DAPI (4′-6′-diamidino-2-phenylindole). (F–H) ALV-J and REV synergistically increased the protein expressions of MSI1 in the nucleus at an MOI of 0.01 at 24 hpi (**F**), 48 hpi (**G**), and 72 hpi (**H**), as detected using WB. (**I and J**) Accumulation of ALV-J RNA and REV RNA due to transient MSI1 expression in DF-1 cells, as detected using qRT-PCR. Relative MSI1 expression levels were detected using WB with an anti-Flag antibody. The association of RNA with immunopurified Flag-MSI1 from DF-1 cells was analyzed using RNA chromatin immunoprecipitation. RNA was extracted from IP material and analyzed using qRT-PCR. Data are presented as mean ± SEM for *n* = 3; each experiment was performed in triplicates. (**K and L**) U3s of ALV-J and REV were the dominant components in different viral RNA gene regions where MSI1 bound, as detected using qRT-PCR. Data are presented as mean ± SEM for *n* = 3; each experiment was performed in triplicates. **P ≤ 0.01 using Student’s *t*-test versus the negative control group. *P ≤ 0.05 using Student‘s *t*-test versus the negative control group.

Previous studies have demonstrated that retroviruses, such as ALV-J and REV, in addition to the Zika virus, PEDV, and CAstV, can increase MSI1 expression within host cells (14). We evaluated viral RNA levels in ALV-J-infected and REV-infected chicken embryo fibroblast (CEF) cells to determine whether MSI1 facilitates the replication of ALV-J and REV. qRT-PCR and WB analyses revealed that MSI1 increased ALV-J and REV RNA and protein levels (Fig. 2A and B). Furthermore, MSI1 knockdown decreased ALV-J and REV mRNA and protein levels (Fig. 2C and D). These results demonstrate that MSI1 is required for the replication of retroviruses. Confocal laser scanning microscopy (CLSM) was used to observe the localization and expression of MSI1 in cells to further uncover the role of host MSI1 in the replication of ALV-J and REV. The predominant localization of MSI1 in normal cells was observed at the periphery of the cytoplasm. When host cells were infected with ALV-J or REV at 72 hpi, the expression of MSI1 in the cytoplasm was significantly upregulated and subsequently accumulated in the nucleus (Fig. 2E). We also examined the nuclear transfer of MSI1 in CEF cells infected with ALV-J or REV at 24 hpi, 48 hpi, and 72 hpi. WB results showed that ALV-J and REV increased the protein expression of MSI1 in the nucleus, thereby promoting its nuclear transfer (Fig. 2F through H). These findings suggest that MSI1 is crucial in the replication and transfer of retroviral nucleic acids.

**Fig 3:**
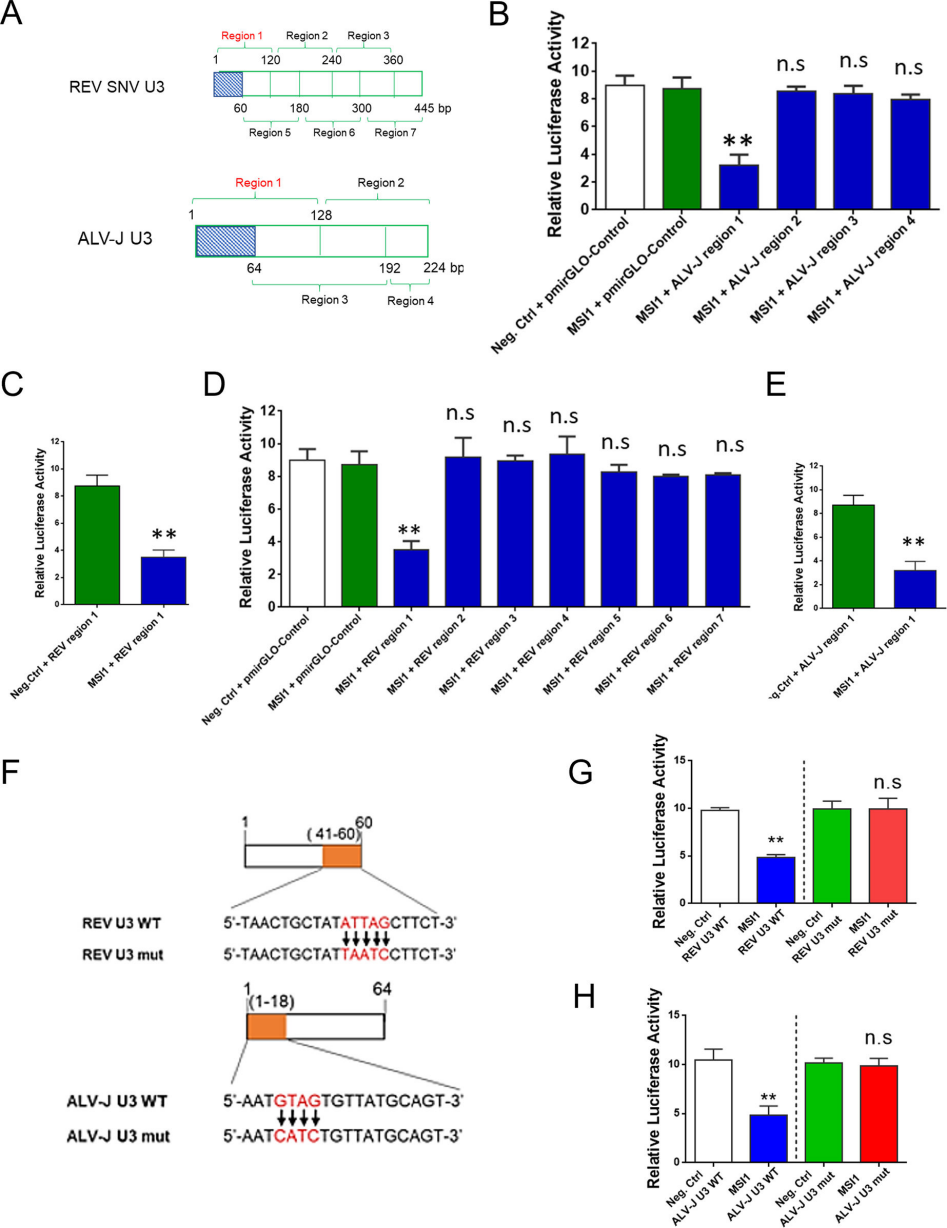
MSI1 targeted U3 regions of ALV-J and REV. (**A**) Strategy for constructing pmirGLO-ALV-J U3 and REV U3 vector plasmids with different U3 sequence regions. (**B and C**) MSI1 or negative control was co-transfected with pmirGLO-Control or ALV-J U3 vector plasmids into 293T cells. Dual-luciferase assay showed that MSI1 significantly inhibited luciferase activities of the ALV-J region 1 vector but not the other region vectors. (**D and E**) MSI1 or negative control was co-transfected with pmirGLO-Control or REV U3 vector plasmids into 293T cells. Dual-luciferase assay showed that MSI1 significantly inhibited luciferase activities of the REV region 1 vector but not the other region vectors. (**F**) Bioinformatics analysis identified one putative MSI1 binding site at the ALV-J U3 and the REV U3. (**G**) Luciferase reporter assays revealed that MSI1 significantly inhibited the activity of the REV U3 reporter. (**H**) Luciferase reporter assays revealed that MSI1 significantly inhibited the activity of the ALV-J U3 reporter. Data are presented as mean ± SEM for *n* = 3, with each experiment being performed in triplicates. **P ≤ 0.01 using Student’s *t*-test versus the negative control group. n.s., not significant.

Furthermore, we used the RNA chromatin immunoprecipitation (ChIP) assay to ascertain whether MSI1 binds directly to viral RNA after transfecting ALV-J-infected or REV-infected cells with Flag-MSI1-expressing plasmids at 48 hpi. The results of RNA ChIP analysis showed that MSI1 directly co-precipitated with viral RNA (Fig. 2I and J). Subsequently, qRT-PCR revealed that the U3 regions of ALV-J and REV were the predominant components of distinct viral RNA sequences bound to MSI1 in cells (Fig. 2K and L). To further elucidate the direct interaction between MSI1, ALV-J U3, and REV U3, we constructed four and seven dual-luciferase vectors based on the ALV-J and REV U3 regions, respectively. Figure 3A shows four different ALV-J U3 regions and seven REV U3 regions. According to the dual-luciferase assay results, MSI1 significantly suppressed the activity of the ALV-J region 1 vector but did not affect the other region vectors (Fig. 3B and C), indicating that the MSI1-targeted ALV-J U3 region lies between nucleotides 1 and 64. Furthermore, MSI1 significantly suppressed the activity of the REV region 1 vector but did not affect the other region vectors (Fig. 3D and E), indicating that the MSI1-targetted REV U3 region lies between nucleotides 1 and 60. Bioinformatics analysis identified one putative MSI1 binding site in ALV-J and REV U3s (Fig. 3F). Luciferase reporter assays revealed that MSI1 significantly inhibited the activity of the ALV-J and REV U3 reporters but not that of the control reporters (Fig. 3G and H). Mutations in the putative MSI1 binding site eliminate the MSI1 inhibitory effect on the reporter activities of REV U3 and ALV-J U3. These findings suggest that MSI1 binds to the U3s of ALV-J and REV in host cells.

### ALV-J, REV, CAstV, and PEDV hijack MSI1 to block the cellular RNase degradation

**Fig 4:**
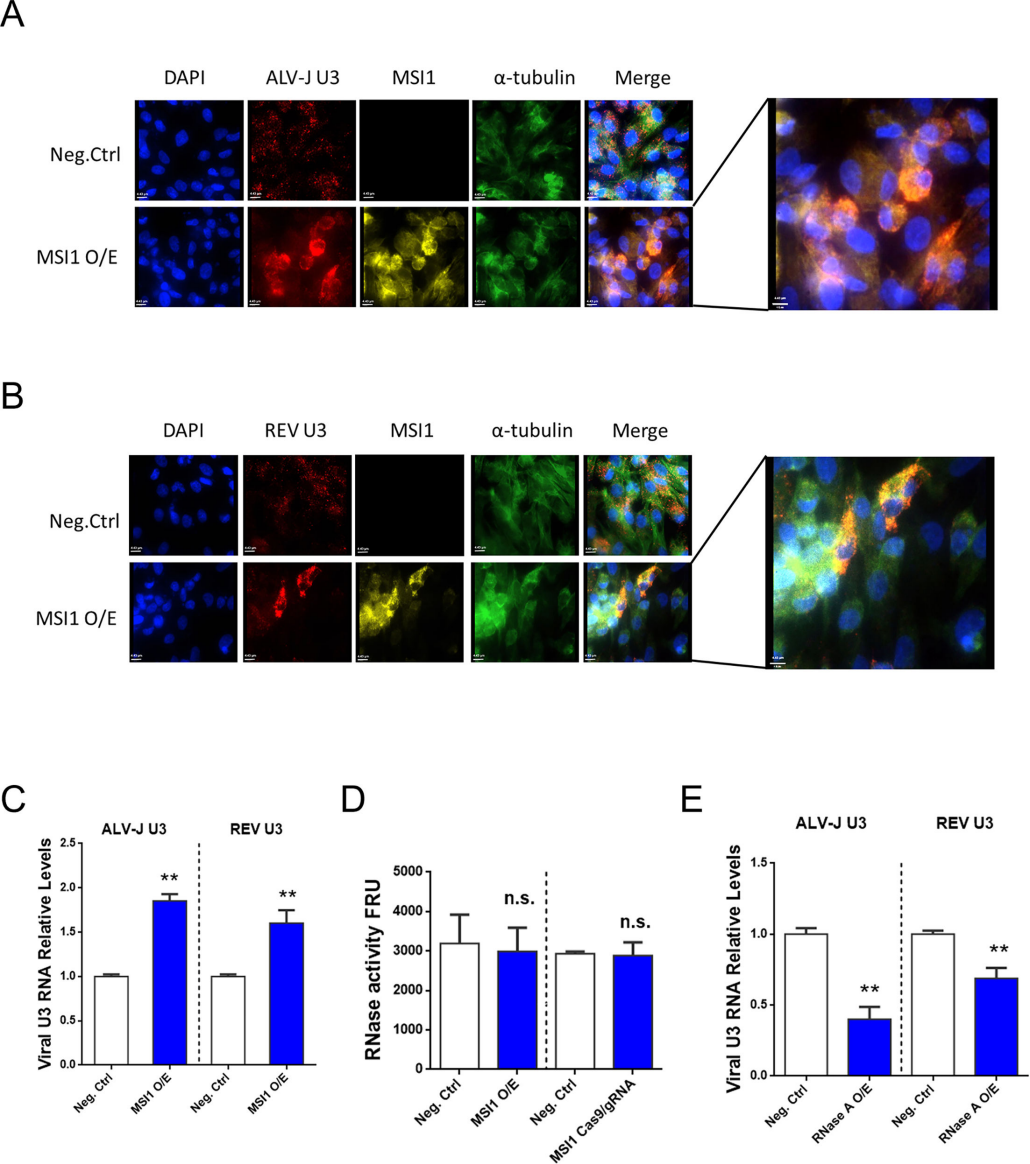
MSI1 prevents the degradation of exogenous ALV-J U3 and exogenous REV U3 by cellular ribonucleases. (**A and B**) CLSM was used to observe the localization and expression of MSI1, ALV-J U3, REV U3, and alpha-tubulin in normal, ALV-J-infected, and REV-infected DF-1 cells at 72 hpi, as detected with anti-Flag antibody, alpha-tubulin monoclonal antibody, ALV-J U3 FISH probe, and REV U3 FISH probe. Nuclei are labeled with DAPI. (**C**) The qRT-PCR results also demonstrated significant upregulations in the expression of viral U3s following MSI1 overexpression. (**D**) RNase activity fluorometric assay indicated that cellular RNase activity remained unchanged, irrespective of the overexpression or interference of MSI1. (**E**) qRT-PCR results showed that the expression of exogenous ALV-J U3 and REV U3 significantly decreased upon overexpression of RNase A. The data are presented as the mean ± SEM for *n* = 3, with each experiment being performed in triplicates. **P ≤ 0.01 using Student‘s *t*-test versus the negative control group. n.s., not significant.

To further verify the effect of MSI1 on viral nucleic acids after binding to retrovirus U3, we initially synthesized exogenous ALV-J U3 (nucleotides 1–64) and REV U3 (nucleotides 1–60) *in vitro*, followed by co-transfection with Flag-MSI1-expressing plasmids into DF-1 cells at 72 hpi. The role of MSI1 in exogenous viral U3 was assessed using CLSM and qRT-PCR. Compared with the negative control, overexpression of MSI1 effectively mitigated the degradation of exogenous ALV-J U3 and REV U3 in the cytoplasm, leading to substantial accumulation of viral U3 at the nuclear periphery and within the cytoplasm, and these accumulations exhibited a high degree of colocalization with alpha-tubulin (Fig. 4A and B). The qRT-PCR results also demonstrated a significant upregulation in the expression of viral U3s following MSI1 overexpression, compared with the negative control (Fig. 4C). RNA degradation in the cytoplasm predominantly relies on RNase activity in the host cell; therefore, we initially investigated whether MSI1 overexpression modulates RNase activity. The results of the RNase activity fluorometric assay indicated that cellular RNase activity remained unchanged irrespective of the overexpression or interference of MSI1 (Fig. 4D). However, when RNase A was overexpressed, the expression of exogenous ALV-J U3 and REV U3 significantly decreased (Fig. 4E), indicating that the binding between MSI1 and U3 effectively protected U3 from RNase degradation.

**Fig 5:**
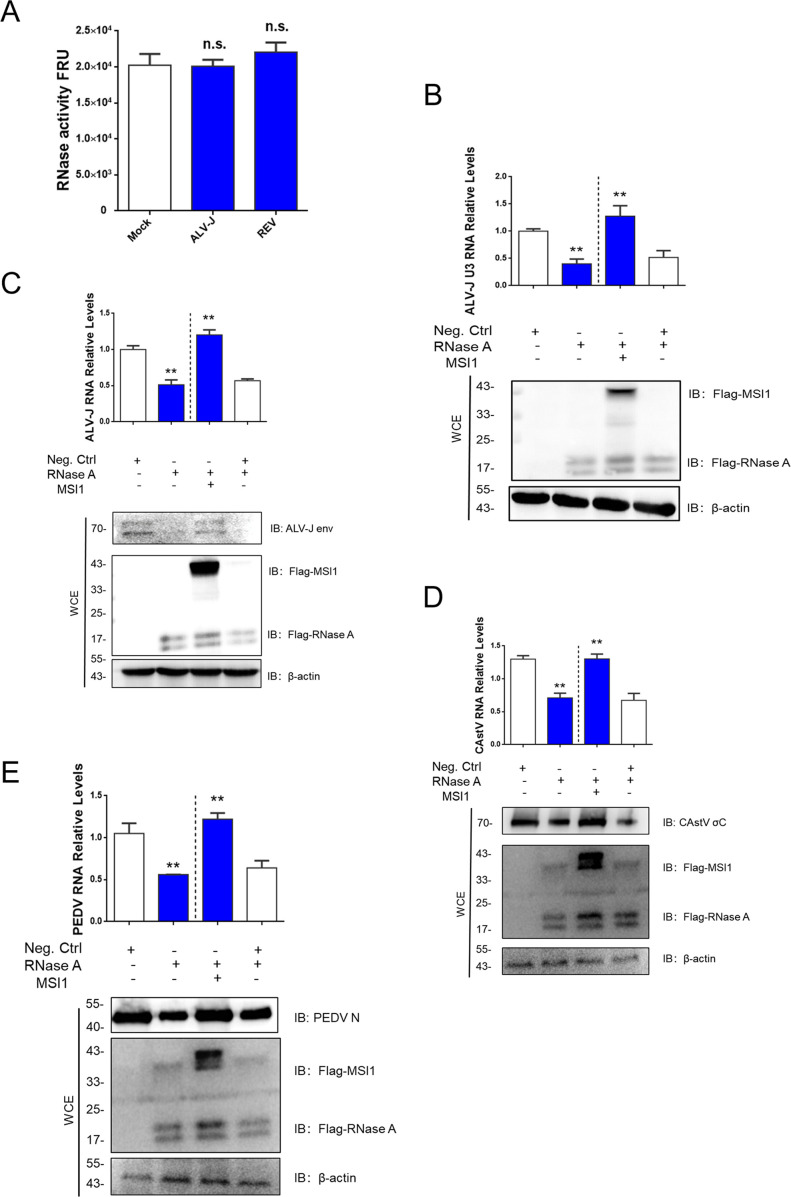
ALV-J, REV, CAstV, and PEDV hijack MSI1 to shield the cellular ribonuclease degradation. (**A**) The RNase activity fluorometric assay indicated that cellular RNase activity remained unaltered, regardless of ALV-J or REV infection. (**B**) qRT-PCR analysis revealed that transfection with Flag-RNase A led to a decrease in ALV-J U3 RNA levels at 48 hpi, while co-transfection with Flag-MSI1 conferred protection to ALV-J U3 RNA against degradation by RNase A. (**C–E**) WB and qRT-PCR analysis revealed that the transfection of Flag-RNase A resulted in decreases in both mRNA and protein levels of ALV-J (**C**), CAstV (**D**), and PEDV (**E**) at 48 hpi, while co-transfection with Flag-MSI1 was found to confer protection to these viruses against degradation by RNase A. The data are presented as the mean ± SEM for *n* = 3, with each experiment being performed in triplicates. **P ≤ 0.01 using Student‘s *t*-test versus the negative control group.

These results were validated using live ALV-J and REV. They indicated that cellular RNase activity remained unaltered regardless of ALV-J or REV infection (Fig. 5A). The qRT-PCR analysis revealed that transfection with Flag-RNase A led to a decrease in ALV-J U3 RNA levels. In contrast, co-transfection with Flag-MSI1 conferred protection to ALV-J U3 RNA against degradation by RNase A (Fig. 5B). Additionally, we corroborated the above results using cells infected with ALV-J, PEDV, and CAstV. Analyses conducted via WB and qRT-PCR revealed that the transfection of Flag-RNase A resulted in decreases in both mRNA and protein levels of ALV-J, PEDV, and CAstV. Co-transfection with Flag-MSI1 was found to confer protection to these viruses against degradation by RNase A (Fig. 5C through E). These results suggest that multiple plus-stranded RNA viruses hijack MSI1 to shield from cellular RNase degradation.

### U3-binding sites of MSI1

**Fig 6:**
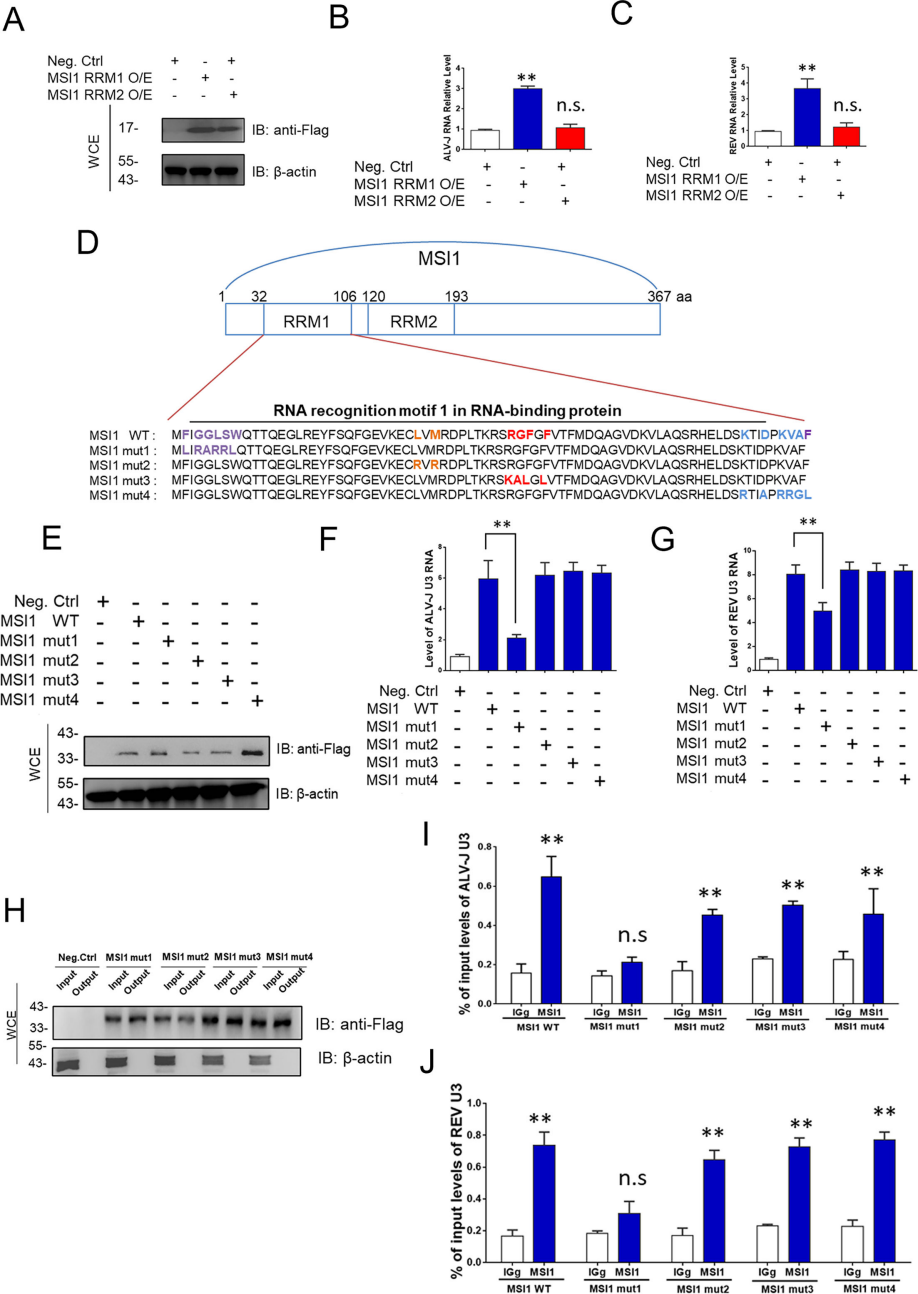
U3-binding regions of MSI1. (**A**) WB analysis demonstrated that RRM1 and RRM2 were successfully transfected into CEFs. (**B and C**) qRT-PCR results showed that only overexpression of MSI1 RRM1 increased ALV-J and REV replication in co-infection at 48 hpi. qRT-PCR data are presented as mean ± SEM and were obtained from three independent experiments (*n* = 3); each experiment contained triplicates. (**D**) Strategy for constructing MSI1 mutants with different RRM1 regions. The original backbone of MSI1 is shown. MSI1 mutants, including MS1 mut1 (F33L, G35R, G36A, L37R, S38R, and W39L), MS1 mut2 (L60R and M62R), MSI1 mut3 (R71K, G72A, F73L, and F75L), and MSI1 mut4 (K98R, D101A, K103R, V104R, A105G, and F106L) were constructed using the Fast site-directed mutagenesis kit. Mutations of amino acid sequences are shown in the same color. (**E**) All MSI mutants were successfully transfected into DF-1 cells co-infected with ALV-J and REV, as detected using WB with the anti-FLAG antibody at 48 hpi. (**F and G**) Among the four mutants, only MSI1 mut1 reversed the increase in ALV-J and REV replication as detected using qRT-PCR at 48 hpi. Data are presented as mean ± SEM for *n* = 3, with each experiment being performed in triplicates. (**H–J**) Among the four mutants, only MSI1 mut1 reversed the accumulation of ALV-J U3 RNA and REV U3 RNA as detected using qRT-PCR. Relative MSI1 mutant expression levels were detected using WB with an anti-Flag antibody. The association of RNA with immunopurified Flag-MSI1 from DF-1 cells was analyzed using RNA ChIP. RNA was extracted from IP material and analyzed using qRT-PCR. Data are presented as mean ± SEM for *n* = 3, with each experiment being performed in triplicates. **P ≤ 0.01 by Student‘s *t*-test versus the negative control group. n.s., not significant.

MSI1 has two N-terminal RNA recognition motifs, RRM1 and RRM2, that mediate binding to motifs located at the 3′UTR of the target gene (22). To determine which RRM promoted viral RNA replication, we measured viral RNA levels in cells co-infected with ALV-J and REV after transfecting RRM1-expressing and RRM2-expressing plasmids at 48 hpi. WB analysis demonstrated that RRM1 and RRM2 were successfully transfected into CEFs (Fig. 6A). The ability of RRM1 to promote viral replication was less than half the function of the complete MSI1 protein; however, qRT-PCR (Fig. 6B and C) analyses revealed that only RRM1 increased the viral RNA levels of ALV-J and REV. Furthermore, we constructed four MSI1 mutants based on its RNA-binding sites (23, 24) and transfected CEFs to detect the RNA levels of ALV-J and REV required to identify the domain sites in MSI1 RRM1 that bind viral U3s. The four MSI1 mutants are depicted in Fig. 6D. WB analysis demonstrated that all MSI1 mutants were successfully transfected into CEFs (Fig. 6E). Notably, based on viral RNA levels, only MSI1 mut1 relieved the promotion of ALV-J and REV replication (Fig. 6F and G), implying that the RNA-binding site (amino acid sequences 2 and 3–7) was the critical domain for binding viral U3s. RNA ChIP analysis revealed that only MSI1 mut1 decreased ALV-J and REV U3 binding (Fig. 6H through J), which is consistent with previous results. These data indicate that MSI1 directly binds to the U3 region of ALV-J and REV through the specific RNA-binding sites of RRM1.

## DISCUSSION

**Fig 7:**
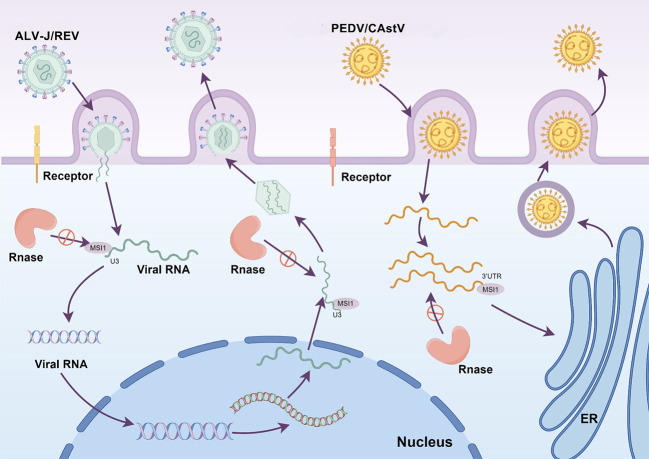
Schematic of the molecular mechanisms by which multiple plus-strand RNA viruses hijack MSI1 to evade cellular ribonuclease degradation. Multiple plus-strand RNA viruses, including ALV-J, REV, CAstV, and PEDV, utilize specific regions such as U3 or 3′UTR to interact with MSI1, thereby protecting their viral RNA from host ribonuclease activity in the cytoplasm. This figure was created using Figdraw.

In the ongoing evolutionary struggle between viruses and their hosts, the utilization of host RBPs by viruses to evade detection and counter host cell defense mechanisms has become prevalent (25, 26). The hijacking of MSI1 by Zika virus through its 3′UTR to enhance viral replication has been demonstrated (11, 12). Furthermore, various plus-strand RNA viruses, including ALV-J, REV, CAstV, and PEDV, harbor MBS. Therefore, exploring the potential of MSI1 to maintain viral replication in other plus-strand RNA viruses that possess MBS in their UTRs is pertinent. We found that plus-strand RNA viruses, including ALV-J, REV, CAstV, and PEDV, hijack MSI1 to evade RNase degradation in the cytoplasm, ultimately completing viral replication (Fig. 7). Notably, the genomes of ALV-J and REV, which belong to the classical retrovirus group, exhibit unique features including RU5 at the 5′ terminus and U3R at the 3′ terminus, distinguishing them from other plus-strand RNA viruses (27–29). MSI1 exhibits a specific binding affinity for the U3 region of ALV-J and REV, thereby inhibiting RNase-mediated degradation of viral RNA in the cytoplasm. These results reveal a critical mechanism through which the plus-stranded RNA virus successfully shields viral RNA from host cellular RNase degradation.

MSI1 is ubiquitous across diverse tissue types and has the capacity for active nucleocytoplasmic shuttling (9, 30). MSI1 is primarily localized within the cytoplasm and is typically absent from the nuclear region in normal cells. Following viral infection, there is a marked increase in MSI1 levels in the cytoplasm, with significant accumulation observed around the perinuclear region, alongside an upregulation of MSI1 expression within the nucleus. Notably, it was observed that these accumulations exhibited a high degree of colocalization with alpha-tubulin, indicating a potential binding interaction between MSI1 and viral RNA within microtubules to shield it from RNase degradation. Furthermore, the release of viral RNA from the nucleus and its transport to different organelles through microtubules are crucial processes (31, 32). Considering that MSI1 is capable of freely shuttling between the nucleus and cytoplasm (33, 34), further research is necessary to ascertain the potential role of its binding to viral RNA in facilitating viral RNA movement.

RNA regulatory mechanisms within host cells are intricately linked to the infection and replication processes of RNA viruses, which may utilize diverse strategies to evade or disrupt host cell RNA regulation, such as detection and degradation by endogenous RNases (35, 36). In this study, intracellular RNase activity remained unchanged irrespective of infection with plus-strand RNA viruses or MSI1 overexpression, suggesting that viral RNA evaded cellular RNase degradation by interfering with intracellular RNase recognition after binding to MSI1. Therefore, it is essential to acknowledge the significant presence of MBSs in the 3′UTR and U3 region of all plus-strand RNA virus strains examined in this study. However, certain plus-strand RNA viruses lack MBSs in their UTRs, suggesting their potential inability to hijack MSI1. The mechanisms by which these plus-strand viral RNAs evade host RNase degradation require further investigation.

In conclusion, this study illustrates that various plus-strand RNA viruses, including ALV-J, REV, CAstV, and PEDV, hijack MSI1 to assist viral replication. Through mechanistic investigations, we determined that these viruses utilize specific regions, such as U3 or 3′UTR, to interact with MSI1, thereby protecting their viral RNA from host RNase degeneration in the cytoplasm. These findings provide valuable insights into the replication strategies of plus-strand RNA viruses and contribute to our overall understanding of their behavior.

## MATERIALS AND METHODS

### Cells, viruses, and plasmids

Primary CEF, DF-1 cells (a spontaneously immortalized CEF cell line), 293T cells, LMH cells (avian leghorn male hepatoma cell line), and Vero cells (African green monkey kidney cells), maintained in the laboratory of animal pathology of Shandong Agriculture University, were cultured in Dulbecco’s modified Eagle’s medium supplemented with 10% fetal bovine serum, 1% penicillin/streptomycin, and 1% l-glutamine, and incubated at 37°C in a 5% carbon dioxide incubator. The stock single-nucleotide variant (SNV) strain of REV at 10^3.2^ 50% tissue culture infectious dose (TCID_50_), NX0101 strain of ALV-J at 10^3.8^ TCID_50_, SD-M strain of PEDV at 10^5.0^ TCID_50_, and SDAU2022-3 strain of CAstV were maintained at the laboratory of animal pathology of Shandong Agriculture University. The REV SNV and NX0101 strains were titrated by limiting the dilution in DF-1 culture to a multiplicity of infection (MOI) of 0.01. The SD-M and SDAU2022-3 strains were titrated by limiting the dilution in the DF-1 culture to an MOI of 0.1.

Bioinformatics analysis software tools and websites, including RNA22, NCBI, ENSEMBLE, and the RNA–protein interaction prediction website (http://pridb.gdcb.iastate.edu/RPISeq/#opennewwindow), were used to analyze and predict the binding sites between MSI1 and viral RNA. ALV-J U3, REV U3, CAstV 3′UTR, and PEDV 3′UTR were cloned downstream of the luciferase reporter gene of the pmirGLO control vector to create wild-type pmirGLO-ALV-J U3 (ALV-J U3 WT), pmirGLO-REV (REV U3 WT), pmirGLO-astrovirus (CAstV 3′UTR WT), and pmirGLO-PEDV (PEDV 3′UTR WT) plasmids, respectively (GenePharma, Shanghai, China). The pmirGLO-ALV-J U3 mutant plasmids (ALV-J U3 mut 1-4), pmirGLO-REV U3 mutant plasmids (REV U3 mut 1-7), pmirGLO-astrovirus mutant plasmid, and pmirGLO-PEDV mutant plasmid were constructed using site-directed mutagenesis. Four and seven dual-luciferase vectors based on the ALV-J U3 and REV U3 regions, respectively, were constructed and purchased from GenePharma (Shanghai, China). Flag-RNase A, Flag-MSI1, MSI1 RRM1, MSI1 RMM2, MSI1Cas9/gRNA, exogenous ALV-J U3 RNA oligos (nucleotides 1–64), and exogenous REV U3 RNA oligos (nucleotides 1–60) were obtained from GenePharma (Shanghai, China). MSI1 mutant plasmids were constructed using a Fast site-directed mutagenesis kit (Tiangen, Beijing, China), following the manufacturer’s instructions.

### Luciferase reporter assays

The ALV-J U3, REV U3, PEDV 3′UTR, and CAstV 3′UTR luciferase reporter plasmids were co-transfected with MSI1 into CEF cells, respectively. Cell lysates were prepared at 48 hpi using the Dual-Lumi luciferase reporter gene assay kit following the manufacturer’s instructions (Beyotime Co., Ltd., Shanghai, China). Renilla luciferase gene activity was standardized using the dual-luciferase reporter assay system (Beyotime Co., Ltd., Shanghai, China).

### Western blotting

Cells were lysed using cell lysis buffer (Beyotime, Beijing, China) and incubated on ice for 5 min. The lysates were resuspended in a sodium dodecyl sulfate loading buffer, boiled for 5 min, loaded, run on a 10% sodium dodecyl sulfate-polyacrylamide gel electrophoresis gel, and transferred onto a nitrocellulose membrane (Solarbio, Beijing, China). Membranes were blocked with 5% skimmed milk at 4°C overnight and probed with anti-MSI1 (rabbit monoclonal ab52865, Abcam, Cambridge, UK), anti-Flag (mouse monoclonal AT0022, Engibody, DE, USA), anti-ALV-J env, anti-REV env, anti-CAstV δC, anti-PEDV N antibodies at 1:2,000, 1:1,000, 1:1,000, 1:1,000, 1:2,000, and 1:500 dilutions, respectively, followed by horseradish peroxidase (HRP)-conjugated goat anti-rabbit secondary antibody (Engibody) or HRP-conjugated goat anti-mouse secondary antibody (Engibody) at a dilution of 1:3,000. Beta-actin was used as the loading control. Anti-ALV-J env monoclonal antibody, anti-REV env monoclonal antibody, and anti-CAstV δC monoclonal antibody were from our lab reserved. The anti-PEDV N polyclonal antibody was kindly provided by Prof. Mingjun Zhu (Northwest A&F University). Protein levels were detected using the enhanced HRP-3,3′-diaminobenzidine tetrahydrochloride chromogenic substrate kit (Tiangen) following the manufacturer’s instructions. The experiments employed two protein ladders, procured from Thermo Fisher Scientific (catalog number 26616) and New Cell and Molecular Biotech (catalog number P9005).

### Quantitative real-time polymerase chain reaction (qPCR)

The specific primer sequences for ALV-J, REV, and glyceraldehyde 3-phosphate dehydrogenase (GAPDH) used in this study are listed in Table 1. Total RNA from CEF cells that had been either mock-infected, mono-infected with ALV-J or REV, or co-infected with ALV-J and REV was isolated using the Tiangen RNeasy mini kit (Tiangen) following the manufacturer’s instructions with optional on-column DNase digestion. RNA integrity and concentration were assessed using agarose gel electrophoresis and spectrophotometry, respectively. RNA (1 µg per triplicate reaction) was reverse-transcribed to cDNA using the Taqman gold reverse transcription kit (Applied Biosystems, Carlsbad, CA, USA). Real-time PCR was performed using SYBR Premix Ex Taq and specific primers. All values were normalized to endogenous GAPDH levels as a control for variation. Assays were performed in triplicate, after which the average threshold cycle (CT) values were used to determine relative concentration differences based on the ΔΔCT method of relative quantization described in the manufacturer’s protocol. Exogenous ALV-J U3 and REV U3 expression was analyzed using the Hairpin-it micro RNA real-time PCR quantitation kit (GenePharma), following the manufacturer’s instructions.

**TABLE 1:** Primer sequences used in qRT-PCR analysis

Gene	Primer sequences	Product (bp)
ALV-J	F: TGCGTGCGTGGTTATTATTTC R: AATGGTGAGGTCGCTGACTGT	144
REV	F: TTGTTGAAGGCAAGCATCAG R: GAGGATAGCATCTGCCCTTT	105
MSI1	F: TTTGTGGGTGGTCTGTCAGTGA R: CGGTGTCGGTTGGTTGTCTTG	116
ALV-J U3	F: TGGTGGAAGTAAGGTGGTATGA R: CTCTGTAATGCGGAACTAAGGA	106
ALV-J U5	F: TTTACCTCCCACCACATTGG R: AGCCTTCCGCTTCATTCAG	91
ALV-J gag	F: GGGAGGAAGTGGGAGAAACAA R: CCGCAATGATAGCAGGATGTG	100
ALV-J pol	F: CCTACAGATATGGCAGACAGAC R: GCAGCAACCGATGTGACA	132
ALV-J env	F: AAGAAGCCGCCAGCAACA R: ATAACCACGCACGCAAGTATC	101
REV U3	F: AGTCTCGCTTCTCGGAATCG R: GTGGTCTGATGCTTGCCTTC	122
REV U5	F: GGTCGCCGTCCTACACATT R: TCTCCTCTCACTGCCAATCTG	101
REV gag	F: CTCCACCTCCATATCCTGAAGT R: CTTGTGCTCCGAACTCTAACC	123
REV pol	F: TCACCTCGTCTCATAACTTGGA R: GTGTATTGGCAGCGTATCGT	182
REV env	F: GGTCTCCGTTCACACTTATCAC R: TGTTCGGCAGTCAATAGGTCTA	156
PEDV N	F: AGATCGCCAGTTTAGCACCA R: GGCAAACCCACATCATCGT	265
CAstV Orf1b	F: ATGTTGGCGTTCCTAATGT R: TCTTCAGCAGCAGCATAC	190
GAPDH	F: GAACATCATCCCAGCGTCCA R: CGGCAGGTCAGGTCAACAAC	132

### FISH and CLSM assay

RNA FISH kit and Cy3-labeled RNA FISH probes of ALV-J U3 and REV U3 were constructed and purchased from GenePharma (Shanghai, China) and used to localize viral U3 following the manufacturer’s protocol. On the day after hybridization with probes of viral U3, samples were removed from the 37°C incubator, washed with 0.1% Tween20 at 42°C for 5 min, washed with 2× saline-sodium citrate (SSC) at 42°C for 5 min, washed with 1× SSC at 42°C for 5 min, incubated with 5% bovine serum albumin for 1 h, and incubated overnight at 4°C with anti-Flag antibody (rabbit monoclonal #AT0502, Engibody, Shanghai, China) and alpha-tubulin monoclonal (B-5-1-2) antibody (Alexa Fluor 488) (#322588, 1:250, Invitrogen, Shanghai, China). Furthermore, the cells were washed thrice with phosphate-buffered saline and incubated with goat anti-rabbit immunoglobulin G heavy and light chains (Alexa Fluor 555) (#ab150086, 1:200, Abcam, Shanghai, China) at 37°C for 1 h. Nuclei were stained with 4′,6-diamidino-2-phenylindole. The slides were mounted with 50% glycerol and examined under a laser confocal microscope (Leica SP8, Leica, Berlin, Germany).

### RNA ChIP assay

The RNA ChIP kit was purchased from Active Motif (Shanghai, China) and used to assess RNA–protein interactions following the manufacturer’s instructions. Cells were fixed using formaldehyde, which cross-links and preserves protein–RNA interactions. The RNA was then sheared into small, uniform fragments using sonication, and a DNase treatment was used to remove residual DNA. MSI1/RNA complexes were immunoprecipitated using anti-Flag (mouse monoclonal AT0022, Engibody, DE, USA) antibodies at 1:100 dilutions. Following immunoprecipitation, cross-linking was reversed, RNA was extracted (using TRIzol extraction), DNase I was added again (to remove residual DNA), and analyzed using RT-qPCR to determine MSI1-bound RNA fragments.

### RNase activity assay

RNase activity fluorometric assay kit was purchased from Beyotime (Shanghai, China) and used to analyze the RNase activity following the manufacturer’s instructions.

### Statistical analysis

Data are presented as mean ± standard deviation(s). A *t*-test and one-way analysis of variance were performed using SPSS v. 13.0 statistical software. Statistical significance was set at *P* < 0.05.

## Data Availability

All data generated or analyzed during this study are included in this published article.
