# Correction for Zhou et al., “Plus-strand RNA viruses hijack Musashi homolog 1 to shield viral RNA from cytoplasmic ribonuclease degradation”

**DOI:** 10.1128/jvi.00333-26

**Published:** 2026-05-05

**Authors:** Defang Zhou, Menglu Xu, Qingjie Liu, Ruixue Xin, Gege Cui, Longying Ding, Xiaoyang Liu, Xinyue Zhang, Tianxing Yan, Jing Zhou, Shuhai He, Liangyu Yang, Bin Xiang, Ziqiang Cheng

## AUTHOR CORRECTION

Volume 99, no. 3, e00023-25, 2025, https://doi.org/10.1128/jvi.00023-25. Figure 2 should appear as shown in this correction. During the proofing process, an operational oversight led to the accidental upload of a draft version of the figure instead of the finalized, formally typeset figure file. The specific corrections to Figure 2 are as follows.

**Fig 2:**
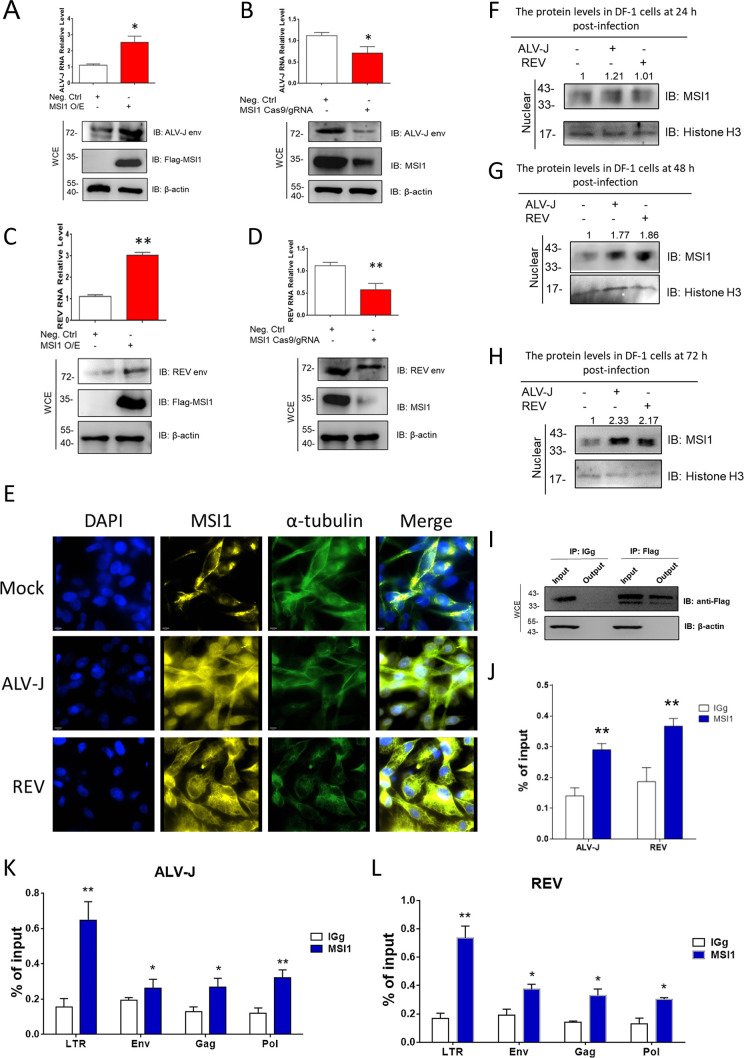


In panels A and B, the data for ALV-J env protein expression levels have been added.

In panel C, the missing label “IB: REV env” has been added.

In panel D, the data for REV env protein expression levels have been added.

In panel E, “tublin” has been corrected to “tubulin.”

In panels F and H, “DF-1 intracellular” has been corrected to “DF-1 cells.”

In panel G, “DF-1 intracellular at 24 h post-infection” has been corrected to “DF-1 cells at 48 h post-infection,” with the corresponding data updated to reflect the validated 48-hour time point results.

The revised figure reflects the data that were reviewed and approved during the peer review process. We apologize for these errors, which did not change the final results.

